# Notch3 signaling promotes colorectal tumor growth by enhancing immunosuppressive cells infiltration in the microenvironment

**DOI:** 10.1186/s12885-023-10526-w

**Published:** 2023-01-16

**Authors:** Kai Huang, Wenwu Luo, Jinmei Fang, Changjun Yu, Guangjie Liu, Xiaodong Yuan, Yun Liu, Wenyong Wu

**Affiliations:** 1 Department of Gastrointestinal Surgery, Department of General Surgery, the First Affiliated Hospital of Anhui Medical University, Hefei, 230022 Anhui China; 2 Department of Pathology, the First Affiliated Hospital of Anhui Medical University, Hefei, 230022 Anhui China; 3 Department of Radiation Oncology, Anhui Provincial Cancer Hospital, The First Affiliated Hospital of USTC, Division of Life Sciences and Medicine, University of Science and Technology of China, Hefei, China; 4 Organ Transplant Center, Department of Hepatobiliary and Transplantation Surgery, The First Affiliated Hospital of USTC, Division of Life Sciences and Medicine, University of Science and Technology of China, Hefei, China; 5 Department of General Surgery, Anhui No.2 Provincial People’s Hospital, Hefei, 230011 China

**Keywords:** Notch3, Colorectal tumor, Tumor microenvironment, Macrophage infiltration

## Abstract

**Background:**

Macrophage infiltration in the tumor microenvironment participates in the regulation of tumor progression. Previous studies have found that Notch signaling pathway is involved in regulating the progression of colorectal cancer (CRC), however, the specific mechanism is still unclear.

**Methods:**

The correlation between Notch signaling pathway and macrophage infiltration was investigated in TCGA database and verified in clinical samples of patients with CRC using immunohistochemistry. Gene Set Enrichment Analysis was used to find out genes related to Notch3 expression. Colony formation assay, and flow cytometry were utilized to test tumor growth and immune cell infiltration in vitro and in vivo.

**Results:**

Using bioinformatics analysis and clinical sample validation, we found that Notch3 was highly expressed in colon tumor tissues compared to adjacent normal tissues, and it participated in regulating the recruitment of macrophages to the tumor microenvironment. Furthermore, we found that the Notch3 expression was positively correlated with the expression of macrophage recruitment-related cytokines in colon tumor tissues. Finally, we demonstrated that depletion of Notch3 had no significant effect on the growth of colon tumor cells in vitro, while, attenuated the growth of colon cancer tumors in vivo. Simultaneous, immunosuppressive cells, macrophages and myeloid-derived suppressor cell (MDSC) infiltration were dramatically reduced in the tumor microenvironment.

**Conclusion:**

Our study illustrated that Notch3 could facilitate the progression of CRC by increasing the infiltration of macrophages and MDSCs to promote the immunosuppressive tumor microenvironment. Targeting Notch3 specifically is a potentially effective treatment for CRC.

**Supplementary Information:**

The online version contains supplementary material available at 10.1186/s12885-023-10526-w.

## Background

Colorectal cancer (CRC) is one of the most predominant malignant tumors in the digestive system, with mortality and morbidity ranking third and fourth worldwide, respectively [1]. In spite of the rapid development of chemotherapeutic and targeted agents, the prognosis for patients with CRC is still unsatisfactory, with less than 10% of the overall five-year survival rate in the late-stage of CRC [2]. The initiation and progression of CRC is complex, and is driven by multiple genetic and epigenetic alterations involving activation of oncogenes and inactivation of tumor-suppressor genes, which promotes intestinal proliferation, survival, invasion, and chemoresistance [3]. Thus, further investigating the underlying mechanisms of the progression of CRC is a crucial issue to identify novel targets for improving treatment efficacy and prognosis of patients with CRC.

Notch signaling is required for the maintenance of intestinal homeostasis. In mammals, there are four Notch receptors and five Notch ligands [4]. Plenty of research has demonstrated that aberrant activation of Notch signaling plays important role in occurrence and development of various cancers, including CRC. In recent years, increasing evidence has shown that overexpression of Notch signaling is observed in CRC and associated with poor prognosis, and inhibition of Notch signaling might be a potential strategy for the prevention and treatment of CRC [5, 6]. Increased Notch3 expression in human CRC tissues and CRC cell lines has also been reported, and is involved in tumor progression and aggressiveness of CRC [7, 8]. In addition, Notch3 participates in the filtration and activity of immune cells within the tumor microenvironment (TME) [9]. It is now accepted that the tumorigenesis is not only mediated by intrinsic properties in cancer cells but also their interactions with non-cancer cells in TME, such as macrophages, lymphocytes, endothelial cells, and stromal fibroblasts. In particular, tumor-associated macrophages (TAMs) have been illustrated to promote growth and metastasis of colon cancer cells [10]. To date, the underlying mechanism of how Notch3 might interact with TME to promote CRC progression is rarely described.

Herein, we firstly demonstrated that Notch3 participated in regulating the recruitment of macrophages in TME. Bioinformatics analysis indicated Notch3 expression was positively correlated with macrophage recruitment pathways and cytokines in CRC. Moreover, we showed that interference with Notch3 itself had no significant effect on the growth of colon cancer cells in vitro, but significantly inhibited the growth of colon tumors in vivo. Further investigation revealed that interfering Notch3 inhibited the infiltration of macrophages. These findings suggest that Notch3 can promote the development of CRC by enhancing the infiltration of macrophage in the TME, which might shed light on the potential role of Notch3 as a therapeutic target in immunotherapy for CRC.

## Methods

### Cell lines and cell culture

MC38, RAW264.7, HCT116 and HEK293T cell lines were obtained from the American Type Culture Collection (Manassas, VA, USA). All cell lines were authenticated by short tandem repeat genotyping before using. The cell lines were cultured in DMEM (Invitrogen/Thermo Fisher Scientific, MA, USA) supplemented with 10% fetal bovine serum (Gibco, Grand Island, NY, USA) and 1% penicillin/streptomycin (P/S) (Beyotime Biotechnology, Jiangsu, China) at 37 °C with 5% CO_2_.

### GSEA (gene set enrichment analysis)

The genes whose expression were positively associated with Notch3 expression in The Cancer Genome Atlas (TCGA)-colorectal carcinoma were picked out using the LinkedOmics browser (http://www.linkedomics.org) [7], which allows the online analysis of TCGA data. Further, we used these genes to do Gene Set Enrichment Analysis. The correlation results were cross-validation using GEPIA [11] (Gene Expression Profiling Interactive Analysis) (http://gepia2.cancer-pku.cn/#index).

### Flow cytometry analysis

Tumors and blood of tumor-carrying mice were collected. Peripheral blood lymphocytes and tumor-infiltrating lymphocytes were isolated, and red blood cells were lysed using Red Blood Cell Lysis Buffer (Beyotime Biotechnology) for 15 mins according to the manufacturer’s instruction manual. Cells were washed and counted using an automated cell counter (Countstar). 1 × 10^6^ cells were blocked with anti-mouse CD16/32 (BioLegend). To identify the percentage of macrophages and myeloid-derived suppressor cells (MDSCs), cells were stained with antibodies: Ax647-CD11b, FITC-Ly-6G, PE-Gr-1,PerCP/Cy7-F4/80 and PerCP/Cy5.5-Ly-6C, purchased from BioLegend company. Flow cytometry acquisition was performed on CytoFLEX (BD Biosciences), and data were analyzed using CytExpert software (2.4.0.28).

### Chemotaxis assay

For analyzing monocyte migration, RAW264.7 cells were seeded (10^5^ cells/ 100 μl DMEM containing 0.1% BSA) onto the top chamber of transwell filters (5-μm; Corning). Filters were placed in a 24-well plate that contains condition medium isolated from the vehicle- or Notch3-depleted MC38 cell lines. 24 hours following incubation, cells in the lower chamber were isolated and counted. Samples were run in triplicates for each group.

### Histology and immunohistochemistry (IHC)

Human CRC tissue samples were obtained from The First Affiliated Hospital of Anhui Medical University. All samples were obtained with informed consent, and the study was approved by the Ethics Committee of Anhui Medical University, as well as the principles expressed in the Declaration of Helsinki. Immunohistochemistry was performed on formalin-fixed, paraffin-embedded human tissue sections using a biotin-avidin method as described before [12]. Sections were developed with DAB and counterstained with hematoxylin. Whole-slide imaging was taken using PANNORAMIC 1000 (3D-HISTECH, Hungary). Quantification of NOTCH3 IHC chromogen intensity was performed as previously described [13]. Briefly, staining intensity (0, 1, 2, 3) and percentage of positive cells among cancer (0–25% recorded as 1, 25–50% as 2, 50–75% as 3 and > 75% as 4) were evaluated by a pathologist. The final immunoreactive scores were calculated by multiplying the two numbers as previously described [13].

### TCGA and GEO data analysis

Clinical data and RNA expression data with normalized RSEM values of the colorectal carcinoma datasets (TCGA-COAD) were retrieved from FireBrowse (http://firebrowse.org). The patients with CRC had associated comprehensive follow-up records, RNA expression data and records of clinical treatment.

### Survival curve

The overall survival of the four Notch receptors was assessed by the TISIDB, a web portal for tumor and immune system interaction, which integrates multiple heterogeneous data types [14].

### Tumor-infiltrating immune cell analysis

TIMER2.0 (http://timer.cistrome.org/), an online tool, which is a comprehensive resource for systematical analysis of immune infiltrates across diverse cancer types, was used to analyze tumor-infiltrating macrophage cells in TCGA-COAD patients [15].

### Western blot

Western blot was performed as previously described [16]. Briefly, cells were lysed on ice for 30 mins using cell lysis buffer (50 mM Tris HCl, pH 7.4, with 250 mM NaCl, 1 mM EDTA, 50 mM NaF, and 0.5% Triton X-100, together with protease inhibitors). Then the lysis was centrifugation at 12000 rpm for 15 mins at 4 °C. The protein concentration of each sample was determined using Bradford Kit (Sangon Biotech. Shanghai, China). For immunoblotting, proteins were resolved by SDS-PAGE and transferred onto polyvinylidene difluoride (PVDF) membranes (Millipore). Immunoblots were developed in Western Lightning Chemiluminescence Reagent Plus (Advansta, Menlo Park, CA, USA).

### Colony formation assay

For colony formation assays, approximately 20,000 cells per well were seeded into 12-well plates and treated with various concentrations of drugs for 72 hrs. Cells were then fixed in 4% formaldehyde for 30 mins and stained with 0.1% (w/v) crystal violet solution.

### Lentivirus production and infection

Lentiviral particles were packaged using HEK293T cells. Briefly, 5 × 10^5^ cells were seed in a 60 mm dish in complete DMEM culture medium 1 day before transfection. On the day of transfection, 18 μl PEI, 3  μg of lentiviral construct as well as 1.5 μg of psPAX2 and pMD2.G were pipet directly into 182 μl serum and antibiotics-free DMEM medium. Then vortexed and added to HEK293T cells in a drop-wise manner 15 mins later. The virus was collected 48 hours later and filtered using non-pyrogenic filters (0.45 mm, Merck Millipore, Billerica, MA, USA). Diluted lentivirus and 8 *μ*g /ml polybrene (Sigma-Aldrich, St. Louis, MO, USA) were added to targeting cells. For stable cell selection, puromycin (1*μ*g/ml) was used.

### 3D spheroid culture

IBAC SR1 3D plates were used to culture MC38 tumor spheroids as reported previously [17]. Briefly, 1 × 10^4^ MC38 cells in DMEM were added to equal volume of Matrigel (Cat#: 356231, Corning) and seeded into IBAC SR1 3D plates after mixture. After 6 days, spheroids of MC38 cells were captured using Olympus microscope equipment with 10× lens.

### In vivo tumor models

All animals were maintained in the house under specific pathogen-free conditions with unrestricted access to food and water for the duration of the experiments. All animal experiments were approved by the Ethics Committee of Anhui Medical University. C57BL/6 and BALB/C nude mice (6–8 weeks old, SLAC Laboratory Animal) were subcutaneously injected with 5 × 10^5^ MC38 and HCT116 cells，respectively. Mice were sacrificed by the use of carbon dioxide inhalation when the tumor volume reached 0.5-1 cm^3^. Tumors were then collected for subsequent experiments.

### Statistical analysis

Data analyses were performed with GraphPad Prism. *P* value was determined using two-way ANOVA for tumor growth, Log-rank test for survival, and two-tailed t-tests for other analyses. *p* < 0.05 was considered as statistically significant.

## Results

### Notch3 expression was significantly positively correlated with macrophage infiltration

**Fig. 1:**
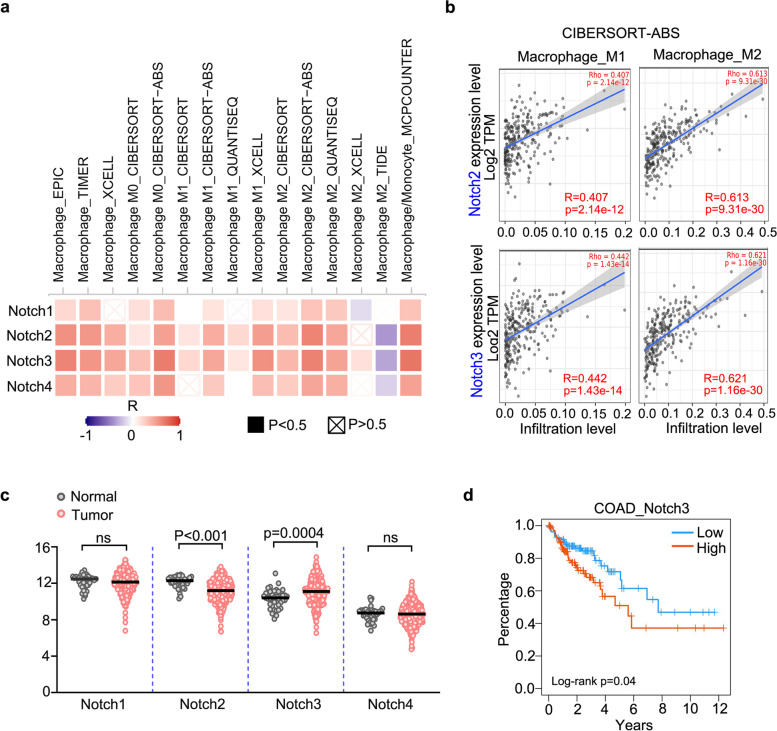
Notch3 expression was significantly positively correlated with macrophage infiltration. (**a**) Summary of the correlation between Notch receptors expression with the infiltration level of different type macrophage cells in colorectal carcinoma. The correlationship was estimated by TIMER2.0 using the TCGA database. (**b)** Representative correlation results analyzed using CIBERSORT-ABS in A. (**c)** The mRNA expression of Notch receptors analyzed using the TCGA database. (**d)** Survival curves of patients with colorectal carcinoma from the TCGA database stratified according to expression levels of Notch3

In order to explore the relationship between Notch signaling pathway and macrophage infiltration in CRC, TCGA databases were used (Fig. 1a). Firstly, it has been shown that Notch receptors expression was positively correlated with the macrophage infiltration in CRC (Fig. 1a). Moreover, similar results were obtained using different calculation methods of immune cell infiltration (Fig. 1a), suggesting the expression of Notch receptors is closely related to the infiltration of macrophages in CRC. By comparing the positive correlation efficient between Notch receptors expression and macrophage infiltration, we found Notch2 and Notch3 were more efficient (Fig. 1b, Supplementary Fig. 1a), indicating these two Notch receptors are more likely to be involved in regulating macrophage infiltration. Then, the expression levels of Notch receptors in colon cancer tumors and normal tissues were analyzed, and we showed that only the level of Notch3 was highly expressed in tumor tissues (Fig. 1c). At the same time, the high expression of Notch3 significantly positively correlated with the poor prognosis of patients with CRC (Fig. 1c, Supplementary Fig. 1b). In summary, we found that Notch3 receptor was positively correlated with macrophage infiltration in CRC as well as the poor prognosis of patients.

### Notch3 expression was positively correlated with the degree of macrophage infiltration in colorectal carcinoma tissue

**Fig. 2:**
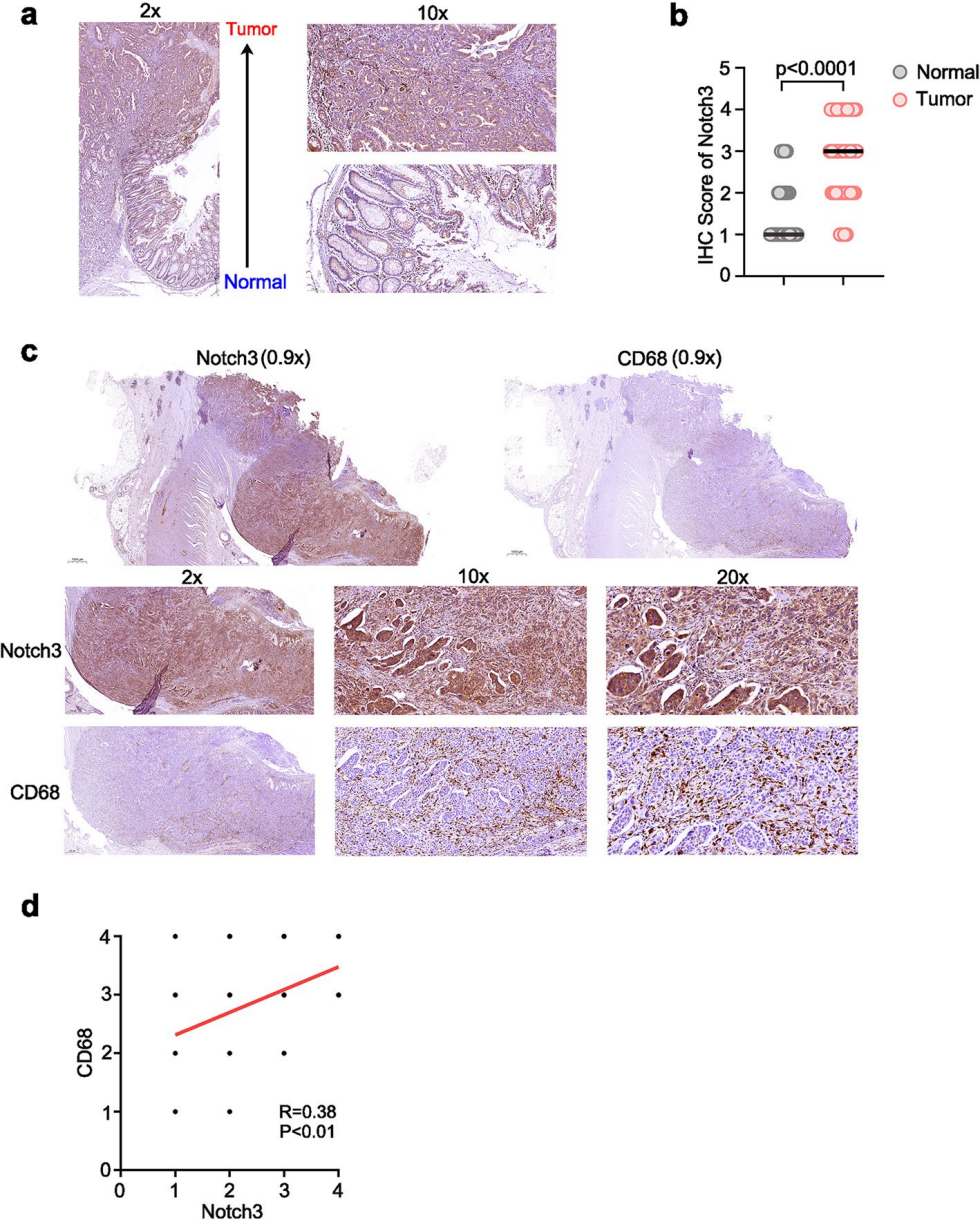
Notch3 expression was positively correlated with the degree of macrophage infiltration in colorectal carcinoma tissue. (**a**) Immunohistochemical staining of colon cancer and adjacent tissue using Notch3 antibody. (**b)** IHC score of Notch3 in colon cancer and adjacent tissue. (**c)** Notch3 and CD68 staining of the colon cancer tissue continuous section from patient #2. (**d)** Analysis of the correlation between Notch3 expression and the percentage of CD68 positive cells

To confirm if Notch3 protein expression was positively correlated with macrophage infiltration, paraffin colorectal carcinoma specimens were collected and stained with Notch3 and CD68, a commonly used macrophage marker. Firstly, to validate the specificity of Notch3 antibody for IHC staining of human tissues, we stained the human HCT116 tumors grown in mice with stable knockdown of Notch3 in comparison to a NTC control (Supplementary Fig. 2a). As shown, the intensity of Notch3 was significantly weakened after knockdown of Notch3 (Supplementary Fig. 2a), which suggested that the antibody of Notch3 is suitable and specificity for IHC. The expression levels of Notch3 were gradually increased as the tumor progressed (Fig. 2a). In addition, Notch3 was highly expressed in colon cancer tissues compared to normal tissues (Fig. 2b, Supplementary Fig. 2b). Next, we stained the serial sections for Notch3 and CD68, respectively, and found the number of CD68-positive cells was significantly increased in the areas with high Notch3 expression (Fig. 2c, Supplementary Fig. 2c). Statistically, a significantly positive correlation was observed between Notch3 expression and macrophage infiltration (Fig. 2d). Taken together, our results indicated that Notch3 protein expression was significantly positively correlated with the degree of macrophage infiltration in colon cancer tumor tissue.

### Notch3 was positive correlated with the expression of macrophage recruitment-related cytokines

**Fig. 3:**
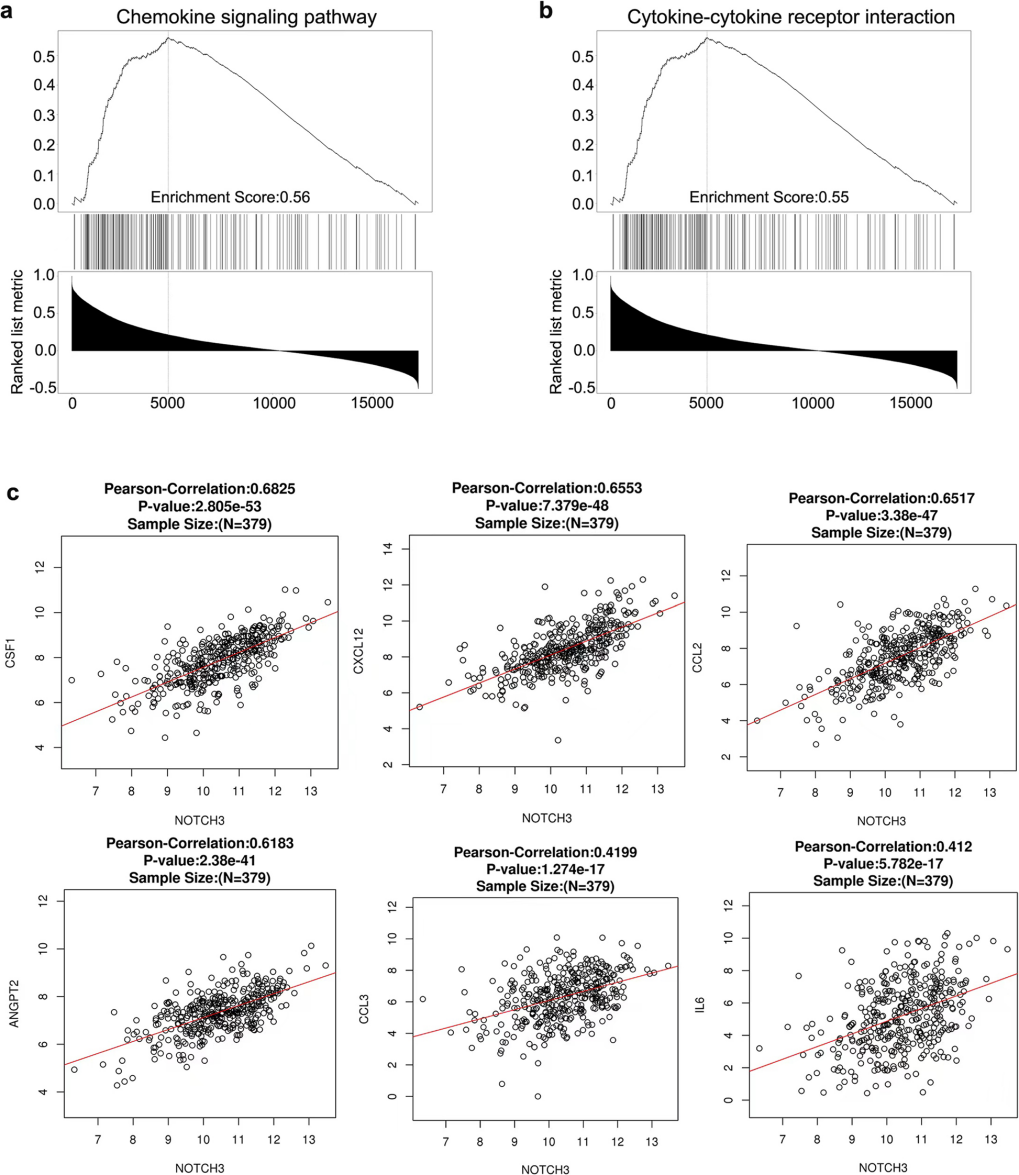
Notch3 was positively correlated with the expression of macrophage recruitment-related cytokines. (**a, b**) GSEA analyze of the genes that correlated with Notch3 using TCGA database. (**c)** The relation between Notch3 and macrophage recruitment-related genes

Next, to further investigate how Notch3 was involved in regulating macrophage infiltration, TCGA database was utilized. Genes that were significantly positively associated with Notch3 expression in colorectal carcinoma were picked out and GSEA enrichment were performed. The results showed that genes related to chemokines and cytokine-receptor binding pathways were significantly enriched (Fig. 3a and b), implying that Notch3 plays an essential role in the macrophage recruitment by regulating the cytokines expression. Then, we analyzed the current known relationship between Notch3 and the expression of macrophage recruitment-related cytokines and found the expression levels of cytokines including CSF1, CXCL12, CCL2 were significantly positively correlated with the expression levels of Notch3, especially CSF1, which is a well-known cytokine involved in promoting macrophage recruitment (Fig. 3c, Supplementary Fig. 3a). To further verify this result, another database-GEPIA was exploited to analyze the above-mentioned cytokines and consistent results were obtained (Supplementary Fig. 3b). Taken together, we demonstrated that Notch3 was significantly positively correlated with the expression of macrophage recruitment-related factors, suggesting that Notch3 can regulate the infiltration of macrophage in tumor tissues by regulating the expression of the above-mentioned cytokines, such as CSF1.

### Interference with Notch3 attenuated the colon tumor growth and decreased macrophage infiltration in vivo

**Fig. 4:**
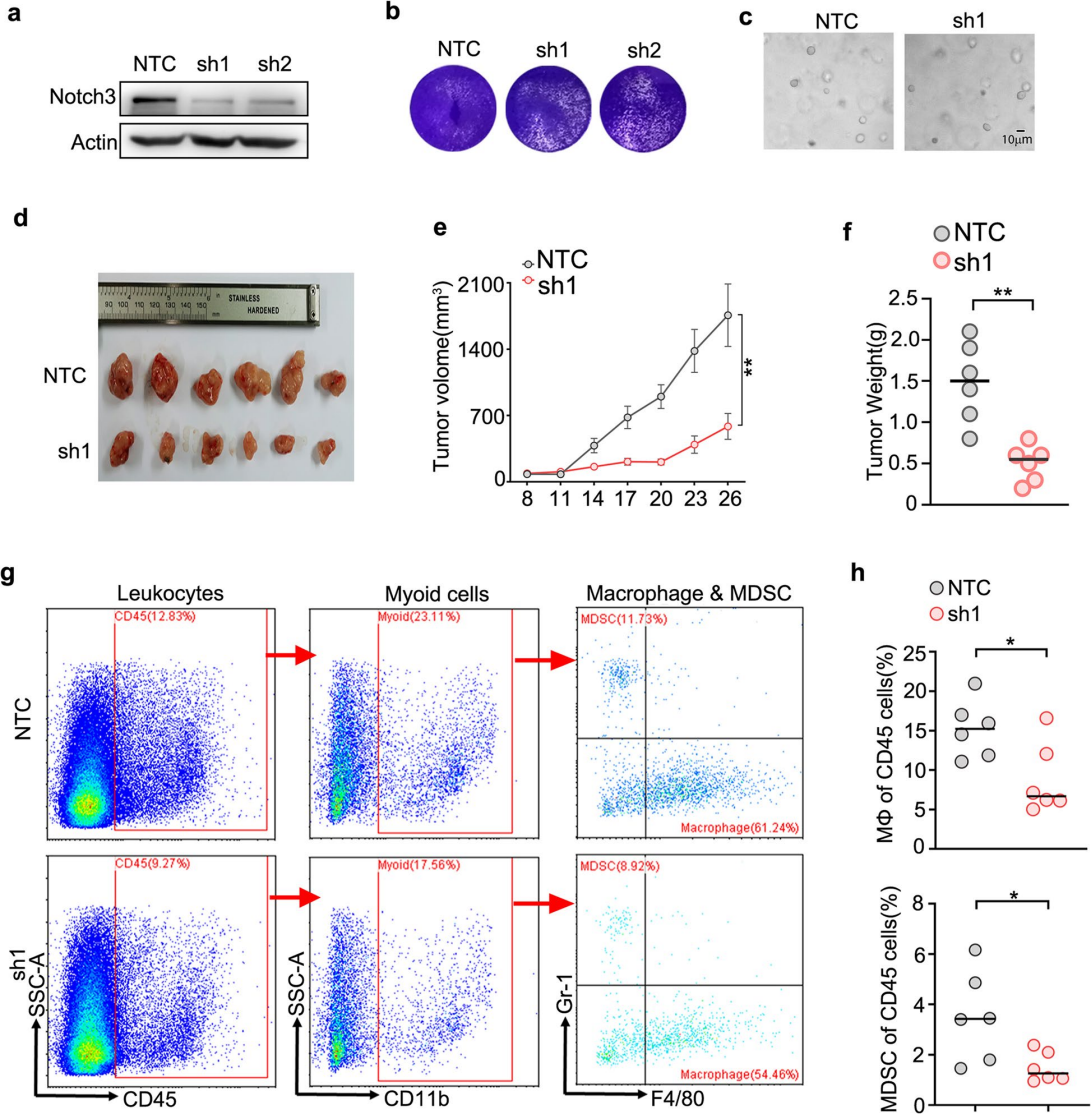
Interference Notch3 attenuate the colon tumor growth and decrease Macrophage infiltration in vivo. (**a**) Knockdown efficiency of Notch3-shRNA in MC38 cells. NTC, non-targeting control. (**b**) Colony formation assay of MC38 cells. (**c**) Spheroid formation assay of MC38 cells. (**d)** MC38 cells transfected with Notch3-shRNA or NTC were subcutaneous injected into C57BL/6 mice, represented tumors were show. (**e**) The proliferation curve of MC38 derived tumors. (**f**) The quantification of tumor weight. (**g**) Flow cytometry analyze of MC38 derived tumors. (**h**) The quantification of the results in g

In order to test whether Notch3 was involved in regulating the recruitment of macrophages in vivo, an MC38 cell line with stable knockdown of Notch3 was constructed (Fig. 4a, Supplementary Fig. 4a). The colony formation assay showed that interference with Notch3 expression in vitro had little effect on cell proliferation (Fig. 4b), which was also verified in 3D growth of MC38 cells (Fig. 4c). Next, we injected control and Notch3 stable knockdown MC38 cells on both sides of C57BL/6 mice. Surprisingly, interference with Notch3 significantly inhibited the growth of transplanted tumors (Fig. 4d-f). The above results indicated that Notch3 mainly promoted the development of CRC by regulating the tumor microenvironment. Furthermore, a flow cytometry experiment was conducted to detect macrophage in the tumor microenvironment of transplanted tumors. The results showed that interference with Notch3 significantly reduced the proportion of macrophage in tumor tissues (Fig. 4g, h, Supplementary Fig. 4b). Meanwhile, the proportion of macrophage was analyzed in the peripheral blood of mice, and no significant changes were observed (Supplementary Fig. 4c). Altogether, our results suggested that Notch3 participated in regulating the recruitment of macrophage in colon cancer, thereby regulating tumor progression.

## Discussion

In the present work, we have shown that the expressions of Notch receptors were positively correlated with the in infiltration of macrophage, among which only Notch3 was highly expressed in colorectal tumor tissues and associated with poor prognosis of patients with CRC. These findings were also confirmed in clinical samples in which Notch3 expression was positively related to the extent of macrophage infiltration. Bioinformatics analysis revealed that Notch3 was found to regulate certain pathways and cytokines involving macrophage recruitment. A notable finding was that inhibition of Notch3 had no influences on colorectal cancer cell proliferation in vitro, whereas interfering with Notch3 significantly decreased tumor growth in vivo. Mechanically, interference with Notch3 significantly reduced the proportion of macrophage in tumor tissue while no obvious changes were detected in peripheral blood of mice, suggesting Notch3 might participate in the regulation of macrophage recruitment and thus promote tumor growth of CRC.

Notch3 is reported to be overexpressed in human CRC and CRC cell lines [18]. Despite the role of Notch3 in CRC has been relatively less investigated, increasing focus is paid on the essential contribution of Notch3 to CRC progression. Ozawa et al. showed significant correlation between nuclear Notch3 expression and tumor recurrence in stage II and stage III CRC [19]. Recently, Varga et al. have indicated that Notch3 expression in CRC was upregulated with tumor stage and correlated to tumor invasion and worse overall survival [20]. In addition, several studies demonstrated that forced Notch3 expression accelerated tumor formation in CRC xenografts while inactivation of Notch3 expression decreased cell proliferation, the clonogenic potential of CRC cells and tumor growth [7, 21, 22]. In consistence with these findings, our study demonstrated that Notch3 plays a crucial role in promoting the growth of CRC. To be noted, we showed that inhibition of Notch3 had no obvious influences on colorectal cancer cell proliferation in vitro. The discrepancy could be attributed to cell-type or context-specific. However, the antitumor effect in vivo has shed lights on the indispensable role of TME which might interact with Notch3 in the development of CRC.

Accumulating evidence has shown the dynamic composition of TME participates in tumor initiation, progression and drug resistance. TAMs as a major component of immune cells in TME play complex roles in tumor development, which can promote or inhibit tumor invasion mediating via interacting with other immune cells and cancer cells. Macrophage infiltration are generally associated with poor clinical outcomes in many solid tumors, including CRC [23]. Using a pre-clinical immunocompetent orthotopic CRC mouse model, Trimaglio et al. revealed a significant role of colon microenvironment in the regulation of CRC immune responses [24]. Recently, a study by Algars et al. focusing on the prognostic role of TAMs in CRC showed that different patterns of macrophage and lymphatic vessels can predict the prognosis according to different stages of CRC [25]. Yin et al. reported that high numbers of TAMs in CRC tissues correlated to chemoresistance by regulating the IL6R/STAT3/miR-204-5p axis, and was associated with poor prognosis in patients with CRC [26].

Notch signaling clearly plays important roles in TAMs, either to promote or suppress tumor growth. The dysregulation of Notch signaling can mediate the interactions between cancer cells and their surrounding TME [27]. Besides, Notch signaling participates in the regulation of macrophage activation and inflammatory processes. Ye et al. demonstrated that Notch and Wnt signaling regulated the differentiation of TAMs in hepatocellular carcinoma. Progression and hepatic metastasis of CRC [28]. Notch signaling has also been described to positively regulate cytokines produced by macrophages which promotes cancer growth and metastasis [29]. Particularly, Notch3 is selectively upregulated during macrophage differentiation and contributes to the pro-inflammatory activation of macrophages [9]. Li et al. recently reported that Notch3 participated in the infiltration and activity of immune cells in TME which contributes to poor prognosis of gastric cancer [30]. Here, we have demonstrated that the expression of Notch3 was positively associated with the level of soluble tumor-derived chemotactic factors, such as CSF1, CXCL12 and CCL2, which are chemotactic stimulus for macrophage recruitment. Macrophage can be recruited to the tumor periphery by CSF-1 produced by CRC cells, and further secret factors to promote cell invasion [31]. CSF1 is required for accumulation of TAM and plays a critical role in macrophage differentiation [32]. In Diffuse large B-cell lymphoma, Huang et al. showed that Notch signaling can regulate the expressions of CSF1 and CCL2, which contributes to TAM modulation and progression [33]. CCL2 as a member of the C-C chemokine family is reported to correlate with the infiltration of TAMs and poor prognosis in many tumors [34]. Chun et al. found the level of CCL2 increased in patients with CRC and neutralization of CCL2 halted tumor progression in a mouse model of inflammation-associated CRC [35]. Shen et al. utilized a breast cancer model and demonstrated that Notch can regulate cancer cell expression of IL1β and CCL2 in vivo experiment, and in vivo models further confirmed an association between Notch-dependent cytokine production and TAM recruitment [36]. It is known that CXCL12 upregulation promotes the recruitment of Tregs and the infiltration of macrophage. Accumulating evidence has shown CXCL12-CXCR4 axis plays a critical role in CRC progression and metastasis [37, 38]. Besides, Zboralski et al. illustrated a key role of CXCL12-CXCR4 axis in conferring resistance to checkpoint inhibitors and blockage of CXCL12 enhanced the efficacy of anti-PD-L1 immunotherapy in a murine model of CRC [39].

To date, there is an urgent need to explore more efficient treatment strategies to improve the outcome of patients with CRC. Regarding the pivotal role of Notch signaling in TME of CRC, targeting Notch with clinically therapeutic agents is a promising CRC treatment approach [40, 41]. Herein, our work elucidates Notch3 as a potential novel target for the treatment of CRC. The associations between Notch signaling and immunotherapeutic efficacy have been investigated, and further explorations of inhibiting Notch signaling is of worthy in clinical applications [42–44]. Based on our findings, the possible utility of targeting Notch3 might be considered as a valuable treatment option in combination with blockage of macrophage as well as MDSC in the future.

## Conclusion

In conclusion, our study elucidated an important role of Notch3 as a regulator in macrophage recruitment and microenvironment in CRC. Notch3 can promote CRC progression via upregulation of macrophage infiltration in TME. Thus, Notch3 might be a promising target in combination with immunotherapy for CRC treatment.

## Supplementary Information

**Additional file 1: Supplementary Fig. 1.** Notch3 expression was significantly positively correlated with macrophages infiltration. a. Representative correlation results analyzed using CIBERSORT-ABS. b. Survival curves of patients with colorectal carcinoma from the TCGA database stratified according to expression levels of Notch1,2,4. **Supplementary Fig. 2.** Notch3 expression was positively correlated with the degree of macrophage infiltration in colorectal carcinoma tissue. a. Immunohistochemical staining of HCT116 with or without Notch3 knockdown derived tumor tissues using Notch3 antibody. b. IHC score standard of Notch3 in colon cancer. c. Notch3 and CD68 staining of the colon cancer tissue continuous section from patient #2. **Supplementary Fig. 3.** Notch3 expression was positively correlated with the macrophage recruitment-related cytokines expression. a. The relation between Notch3 and macrophage recruitment-related genes analyzed using LinkedOmics database (http://www.linkedomics.org/login.php). b. The relationship between Notch3 and macrophage recruitment-related genes analyzed using GEPIA2 database (http://gepia2.cancer-pku.cn/#index). **Supplementary Fig. 4.** Interference Notch3 attenuated the colon tumor growth and decreased macrophage infiltration in vivo. a. Notch3 staining of NCT and Notch3-shRNA in mice tumor tissues. b. Gating strategy for flow cytometry. c. Flow cytometry analysis of blood from MC38 tumors carrying mice. d. The quantification of the results in b.

## Data Availability

The public datasets could be downloaded at http://www.linkedomics.org (cohort: TCGA) and http://gepia2.cancer-pku.cn/#index (cohort: GEPIA). Other data analyzed during the current study are available from the corresponding author on reasonable request.

## Abbreviations

CRC: Colorectal cancer
IHC: immunohistochemistry
TAMs: tumor-associated macrophages
TCGA: The Cancer Genome Atlas
TME: tumor microenvironment.
