# Supplementary material for: Notch3 signaling promotes colorectal tumor growth by enhancing immunosuppressive cells infiltration in the microenvironment
Source: BMC Cancer. 2023 Jan 16;23:55. doi: 10.1186/s12885-023-10526-w (PMC9843853; doi:10.1186/s12885-023-10526-w)
Supplement: Supplementary file 1 — Additional file 1: Supplementary Fig. 1. Notch3 expression was significantly positively correlated with macrophages infiltration. a. Representative correlation results analyzed using CIBERSORT-ABS. b. Survival curves of patients with colorectal carcinoma from the TCGA database stratified according to expression levels of Notch1,2,4. Supplementary Fig. 2. Notch3 expression was positively correlated with the degree of macrophage infiltration in colorectal carcinoma tissue. a. Immunohistochemical staining of HCT116 with or without Notch3 knockdown derived tumor tissues using Notch3 antibody. b. IHC score standard of Notch3 in colon cancer. c. Notch3 and CD68 staining of the colon cancer tissue continuous section from patient #2. Supplementary Fig. 3. Notch3 expression was positively correlated with the macrophage recruitment-related cytokines expression. a. The relation between Notch3 and macrophage recruitment-related genes analyzed using LinkedOmics database (http://www.linkedomics.org/login.php). b. The relationship between Notch3 and macrophage recruitment-related genes analyzed using GEPIA2 database (http://gepia2.cancer-pku.cn/#index). Supplementary Fig. 4. Interference Notch3 attenuated the colon tumor growth and decreased macrophage infiltration in vivo. a. Notch3 staining of NCT and Notch3-shRNA in mice tumor tissues. b. Gating strategy for flow cytometry. c. Flow cytometry analysis of blood from MC38 tumors carrying mice. d. The quantification of the results in b. [file 12885_2023_10526_MOESM1_ESM.docx]

**Supplementary Materials**

**Additional file 1:**

**Supplementary Figure 1. Notch3 expression was significantly positively correlated with macrophages infiltration. A.** Representative correlation results analyzed using CIBERSORT-ABS. **B.** Survival curves of patients with colorectal carcinoma from the TCGA database stratified according to expression levels of Notch1,2,4.

**Supplementary Figure 2. Notch3 expression was positively correlated with the degree of macrophage infiltration in colorectal carcinoma tissue. A.** IHC score standard of Notch3 in colon cancer. **B.** Notch3 and CD68 staining of the colon cancer tissue continuous section from patient #2.

**Supplementary Figure 3. Notch3 expression was positively correlated with the macrophage recruitment-related cytokines expression. A.** The relation between Notch3 and macrophage recruitment-related genes analyzed using LinkedOmics database (http://www.linkedomics.org/login.php). **B.** The relationship between Notch3 and macrophage recruitment-related genes analyzed using GEPIA2 database (http://gepia2.cancer-pku.cn/#index).

**Supplementary Figure 4. Interference Notch3 attenuated the colon tumor growth and decreased macrophage infiltration *in vivo*. A.** Notch3 staining of NCT and Notch3-shRNA in mice tumor tissues. **B.** Gating strategy for flow cytometry. **C.** Flow cytometry analysis of blood from MC38 tumors carrying mice. **D.** The quantification of the results in B.
